# A morphological method for ammonia detection in liver

**DOI:** 10.1371/journal.pone.0173914

**Published:** 2017-03-20

**Authors:** Virginia Gutiérrez-de-Juan, Sergio López de Davalillo, David Fernández-Ramos, Lucía Barbier-Torres, Imanol Zubiete-Franco, Pablo Fernández-Tussy, Jorge Simon, Fernando Lopitz-Otsoa, Javier de las Heras, Paula Iruzubieta, María Teresa Arias-Loste, Erica Villa, Javier Crespo, Raúl Andrade, M. Isabel Lucena, Marta Varela-Rey, Shelly C. Lu, José M. Mato, Teresa Cardoso Delgado, María-Luz Martínez-Chantar

**Affiliations:** 1 CIC bioGUNE (Center for Cooperative Research in Biosciences), Centro de Investigación Biomédica en Red de Enfermedades Hepáticas y Digestivas (CIBERehd), Derio, Bizkaia, Spain; 2 Division of Pediatric Metabolism, University Hospital of Cruces, BioCruces Health Research Institute, University of the Basque Country, UPV/EHU, Barakaldo, Bizkaia, Spain; 3 Gastroenterology and Hepatology Department, Marqués de Valdecilla University Hospital, Centro de Investigación Biomédica en Red de Enfermedades Hepáticas y Digestivas (CIBERehd), Santander, Spain; 4 Infection, Immunity and Digestive Pathology Group, Research Institute Marqués de Valdecilla (IDIVAL), Santander, Spain; 5 Department of Gastroenterology, Azienda Ospedaliero-Universitaria & University of Modena and Reggio Emilia, Modena, Italy; 6 Unidad de Gestión Clínica de Aparato Digestivo, Servicio de Farmacología Clínica, Instituto de Investigación Biomédica de Málaga-IBIMA, Hospital Universitario Virgen de la Victoria, Universidad de Málaga, Centro de Investigación Biomédica en Red de Enfermedades Hepáticas y Digestivas (CIBERehd), Málaga, Spain; 7 Division of Digestive and Liver Disease, Cedars-Sinai Medical Center, Los Angeles, California, United States of America; University of Navarra School of Medicine and Center for Applied Medical Research (CIMA), SPAIN

## Abstract

Hyperammonemia is a metabolic condition characterized by elevated levels of ammonia and a common event in acute liver injury/failure and chronic liver disease. Even though hepatic ammonia levels are potential predictive factors of patient outcome, easy and inexpensive methods aiming at the detection of liver ammonia accumulation in the clinical setting remain unavailable. Thus, herein we have developed a morphological method, based on the utilization of Nessler´s reagent, to accurately and precisely detect the accumulation of ammonia in biological tissue. We have validated our method against a commercially available kit in mouse tissue samples and, by using this modified method, we have confirmed the hepatic accumulation of ammonia in clinical and animal models of acute and chronic advanced liver injury as well as in the progression of fatty liver disease. Overall, we propose a morphological method for ammonia detection in liver that correlates well with the degree of liver disease severity and therefore can be potentially used to predict patient outcome.

## Introduction

Ammonia is one of the main products of nitrogen metabolism that is usually transported from muscle and other peripheral tissues to the liver to be converted to urea by the urea cycle and excreted by the kidneys in the form of urine. Hyperammonemia is a life-threatening metabolic condition characterized by elevated levels of ammonia and as be known for a long time to be a common event in acute and chronic liver injury [1]. Indeed, admission ammonia level was shown to be a predictive factor of mortality in acute liver patients [2] whereas hyperammonemia was previously associated with increasing Child-Pugh grade of liver cirrhosis [3]. In agreement with these line of evidence, several ammonia-lowering therapies have been associated with improvements in acute liver failure [4] and chronic liver disease outcomes, both in experimental models [5, 6] and in the clinical setting [7–9]. Hyperammonemic crisis play an important role in the development of hepatic encephalopathy [10], a common neuropsychiatric abnormality, which further complicates the already complicated clinical course of these patients [11, 12]. Furthermore, increased ammonia can disturb many organ and cell types leading to dysfunction [13–15]. Indeed, it has been recently shown that ammonia lowering reverses sarcopenia of cirrhosis by restoring skeletal muscle proteostasis [16].

Accurate prognosis in patients with liver disease is difficult but critical because liver transplantation is potentially lifesaving. Under these circumstances, ammonia levels reflecting disturbed liver metabolism have been suggested as predictive markers of patient outcome [17–19]. On the other hand, the relevance of the accumulation of hepatic ammonia in non-alcoholic fatty liver disease (NAFLD) and in the progression from hepatic steatosis to non-alcoholic steatophepatitis (NASH) is not completely understood to date. Current methods for hepatic ammonia quantification include several indirect and imprecise methods, such as arterial blood gas analysis, measurement of serum amino acid levels, urinary ketone tests, and the direct quantification of ammonia in fluids and tissue extracts. Whereas blood ammonia levels are often used to evaluate liver disease progression, the direct measurements of reliable ammonia in the blood are tricky in clinical chemistry as delays in either transportation to the laboratory after collection or before completion of analysis are possible confounding factors [20]. Also, blood ammonia levels reflect whole-body homeostasis of nitrogen metabolism and not only liver ammonia metabolism. Importantly, patients with liver disease are often subjected to liver biopsies for accurate diagnosis and therefore liver biopsies are not limiting in these patients. In spite of this, the colorimetric kits currently used for ammonia determination in tissue extracts are rather expensive and sensitive to ammonia sources present in the environment that necessitates working in a glovebox or a negative air pressure area, which is not always available at all the clinical facilities. Moreover, prolonged tissue storage, even at -80°C, may affect the stability of samples resulting in ammonia readings lower than expected. Therefore, the implementation of novel and reliable inexpensive methods to routinely assess ammonia in biological samples and in the clinical setting, especially focusing on liver tissue biopsies, is needed.

Herein, we have developed a morphological method to assess ammonia in biological tissues by using Nessler´s reagent, an alkaline solution of mercury (II) iodide in potassium iodide. When in contact with ammonia, Nessler´s reagent forms an orange solution, which becomes a brown precipitate at higher concentrations of ammonia. Indeed, we have validated our assay to detect ammonia against a commercial kit for ammonia quantification in an array of different mouse tissues. Moreover, we have compared liver samples from injured and healthy animals and translated our findings into the clinical setting, showing the well-described accumulation of ammonia in the liver of cirrhotic and drug-induced liver injury (DILI) patients as well as the involvement of ammonia accumulation in association with steatosis and the pathogenesis of NASH.

## Methods

### Human and animal samples

Adult male mice were housed at the Animal unit at CIC bioGUNE (AAALAC-accredited facility). C57BL/6J mice were purchased from Jackson Laboratories. All animals were housed in a controlled environment under a light cycle with free access to water and food. Animal procedures were approved by the CIC bioGUNE Animal Care and Use Committee according to the criteria outlined in the “Guide for the Care and Use of Laboratory Animals” prepared by the National Academy of Sciences and published by the NIH (publication 86–23 revised 1985). As an experimental model of liver injury, C57BL/6 male mice were subjected to bile-duct ligation (BDL) surgery and sacrificed either at 10 or 14 days after surgery. Bile-duct ligation surgery was performed as previously described [21, 22] in animals under isoflurane anesthesia. After surgery, animals were continuously monitored until 72-hours after and given non-steroidal anti-inflammatory drugs each 24-hours. In addition, we have used a transgenic animal model of chronic biliary liver disease, the Mdr2 (Abcb4)(^-/-^) mouse [23] and compared to its wild-type controls. As an experimental model of DILI, adult C57BL/6 male mice were given an intraperitoneal bolus of 360mg/kg acetaminophen (paracetamol, N-acetyl-p-aminophenol; APAP) (Sigma-Aldrich, St Quentinfallavier, France) dissolved in phosphate buffer saline and sacrificed after 48h. Control animals were given buffer alone control. As an animal model of hepatic steatosis, C57BL/6 mice were fed a high fat diet (EF D12492, 60% from fat, ssniff^®^, Soest, Germany) for 20 weeks and as an animal model of progression from NAFLD to NASH, mice were fed a choline deficient diet with 0.1% methionine (A02082006, Research Diets, Inc., New Jersey, USA). At least n = 5 animals were used for each experimental group.

Human liver specimens include biopsies of cirrhotic livers from the Marqués de Valdecilla Hospital, Santander, Spain (n = 6) from patients that underwent liver transplantation. Patients had been diagnosed with cirrhosis for 4 ± 1 years with Child-Pugh score of either B or C and an average MELD score of 18 ± 1. In addition, liver biopsy samples from 7 patients retrieved from the Spanish DILI Registry and diagnosed of idiosyncratic drug-induced liver injury related to isoniacid, rifampicin and pyrazinamide therapy (cholestatic damage), transilat (an investigational drug) (mixed liver injury), cloxacillin (cholestatic), and fluvastatin, interferon alfa-2a, naproxen and isoflavone with hepatocellular damage were included. There were 4 women, mean age of 46 years (range 16 to 67 years) and a median duration of treatments of 33 days (14, 25th percentile, 150, 75th percentile). Mean biochemical parameters at the time of DILI diagnosis were 5.7mg/dL of TB (range: 0.7–12), 7.8xULN of AST (range: 2.6–26), 8.9xULN of ALT (range: 3–28), 8.3xULN of GGT (range: 2–36) and 2.6xULN of ALP (range: 0.5–7). The mean time elapsed from DILI recognition to liver biopsy was 2 months, in one patient two biopsies were performed, the first at one month from DILI onset and the last at 1 one year and 9 months. Liver biopsies showed two cases of focal necrosis, one of these with mild portal fibrosis, one with sinusoidal collapse, one with granulomatous reaction, one with cholestasis, one with regenerative changes presenting with alterations of Kupffer cells and one with chronic hepatitis and moderate fibrosis. Finally, liver biopsies of well-characterized obese non-alcoholic fatty liver disease (NAFLD) patients subjected to bariatric surgery from the Valdecilla Hospital, Santander, Spain, were used (n = 33). Histological scoring was performed according to the NASH Clinical Research Network criteria [24]. Healthy human liver samples from organ-transplant donors were used as controls. All patients gave informed consent to all clinical investigations, which were performed in accord with the principles and guidelines embodied in the 1975 Declaration of Helsinki in *a priori* approval by the institutional human research review committee. The Institutional committees approving the experiments were: the ethics committee from Marqués de Valdecilla Hospital, Santander, Spain and from the Hospital Universitario Virgen de la Victoria, Málaga, Spain.

### Mouse tissue collection and sample handling

At the end-point of each experiment animals, 200 μl of retro-orbital blood was collected from anesthetized animals and afterwards euthanasia was performed by cervical dislocation. For tissue array, necropsy of animals was performed according to the methods described by the Jackson laboratory. The order of extraction was the following according to the order of tissue autolysis: liver, pancreas, kidney, brain and muscle. Samples extracted were collected in Optimal Temperature Compound (O.C.T) and flash frozen fresh until further use. Before staining, O.C.T. included samples were cut using cryostat (Leica Biosystems CM1520). Alternatively, biological tissues were fixed in 10% neutral buffered formalin (NBF) (20:1 v/v). The 10% NBF was replaced by fresh fixative to eliminate blood and feces from the fixative. After 24h of fixation, tissues were placed in 50% alcohol and processed and embedded in paraffin with an automated tissue processor (Leica Biosystems TP1020, Barcelona, Spain) and an embedding center (Leica Biosystems EG1150H, Barcelona, Spain). Tissues were oriented properly previous to the final embedding in paraffin.

### Tissue array construction

Fixed tissues were sectioned at 5 μm in a standard microtome (Leica Biosystems, RM2245, Barcelona, Spain). Sections were dewaxed in two 10-minutes changes of histoclear and hydrated from 100% alcohol to distilled water through 5-minutes changes of graded alcohols. Then sections were stained 5 minutes in Harris hematoxylin, differentiated in 0.5% hydrochloric acid for 3 seconds and washed with running tap water for 5 minutes. Sections were stained with aqueous eosin solution for 15 minutes and dehydrated through graded alcohols. Finally, samples were cleared with histoclear for 10 minutes (two changes of 5 minutes each) and permanently mounted with DPX mounting medium. Representative areas of the different tissues were selected under a light microscope for tissue array construction using a permanent marker. Afterwards, the hematoxylin-eosin (H&E) stained sections were placed over the donor block for spot selection with the punch of the tissue arrayer. Using the manual Beecher tissue arrayer spots of the array were selected with the 0.6 mm punch and placed in the recipient paraffin block. Once all the spots were placed in the recipient, the block was warmed at 60°C during 10 minutes to melt the new spots with the paraffin of the recipient. 5 μm serial samples of the tissue array were sectioned for further analysis.

### Nessler´s staining of biological tissues

Five μm samples of the paraffin tissue array or O.C.T. included frozen samples were used for ammonia stain with Nessler´s reagent according to the following protocol. Frozen samples were fixed in NBF 10% for 15 min whereas paraffin embedded sections were dewaxed in two 10 minutes changes of histoclear and hydrated from 100% alcohol to distilled water through 5 minutes changes of graded alcohols. Then sections were placed in a humid chamber in a fume hood (Nessler´s reagent is highly toxic) and surrounded with a hidrofuge pap pen to avoid loss of liquid during the procedure. Samples were placed in distilled water to avoid dehydratation during the staining process.

To begin with the procedure, water was drained and Nessler´s reagent was gently shaken to prevent precipitations. Then, sections were incubated exactly 5 minutes with 100 mL of Nessler´s reagent each and washed for 10 seconds with sterile distilled water stirring the samples gently to develop the color. Samples were counterstained with Mayers haematoxylin, washed with running tap water and dehydrated briefly before clearing with histoclear (two changes of 20 seconds each). Samples were mounted with DPX permanent mounting medium.

Nessler´s reagent is an aqueous yellow pale solution of potassium iodide, mercuric chloride, and potassium hydroxide used for ammonia determination. This solution becomes a darker yellow in the presence of ammonia. At higher concentrations of ammonia, a brown precipitate may form according to the following reaction [25]:

$$\begin{array}{l} {\text{NH}}_{4}^{+}+2{\left[{\text{HgI}}_{4}\right]}^{2-}+4{\text{OH}}^{-}\;\to \text{HgO}.\text{Hg}\left({\text{NH}}_{2}\right)\text{I}↓+\;7{\text{I}}^{-}+\;3{\text{H}}_{2}\text{O}\\ \;\;\;\;\;\;\;\;\left(\text{pale yellow}\right)\;\;\;\;\;\left(\text{orange-brown}\right) \end{array}$$

### Image analysis

Image CellProfiler open-source software was used for automated image analysis [26]. Protocols for image analysis quantification of ammonia in samples are included. The software uses pipelines, which is a series of modules easily added or removed from the protocol. Each module performs a specific task on the image. For this pipeline, color images were firstly converted to grayscale. Then, as a conversion method images were split into HSV and HUE channels, saturation and value channels respectively. The output value images were inverted and selected, with a 0.7 threshold for all images. Then, a binary image was obtained with the selected area of stained ammonia. The next module of the pipeline measures the image area occupied. Finally, results were exported to a spreadsheet for further analysis.

### Ammonia Score (AS)

To be able to compare ammonia quantifications between different laboratories and hospital facilities we propose a scoring system, Ammonia Score (AS), as follows: at least 3 samples from healthy livers either human or mouse are used as controls and used for normalization of the rest of the samples; samples are normalized to the average values of these samples and their score classified according to the following criteria (Area ratio between 0-2- Score 1, area ratio between 2-10- Score 2, area ratio between 10-20- Score 3, area ratio between 20-30- Score 4, area ratio between 30-40- Score 5, area ratio >40- Score 6). Data are represented in a color scale and * *p*<0.05 between study groups indicated.

### Ammonia assay kit

Tissue ammonia assay was performed following the instructions of the kit supplier (abcam, Cambridge, United Kingdom). It is a colorimetric assay where ammonia is converted to a product that reacts with the OxiRed probe to generate color (λmax = 570 nm) that can be quantified by using a spectrophotometric plate reader. On the other hand, for ammonia serum quantification we have used a commercial available kit provided by Sigma-Aldrich (St. Louis, MO, USA) based on the *o*-phthaladehyde method.

## Results

### Ammonia distribution in biological tissues of healthy rodents

**Fig 1:**
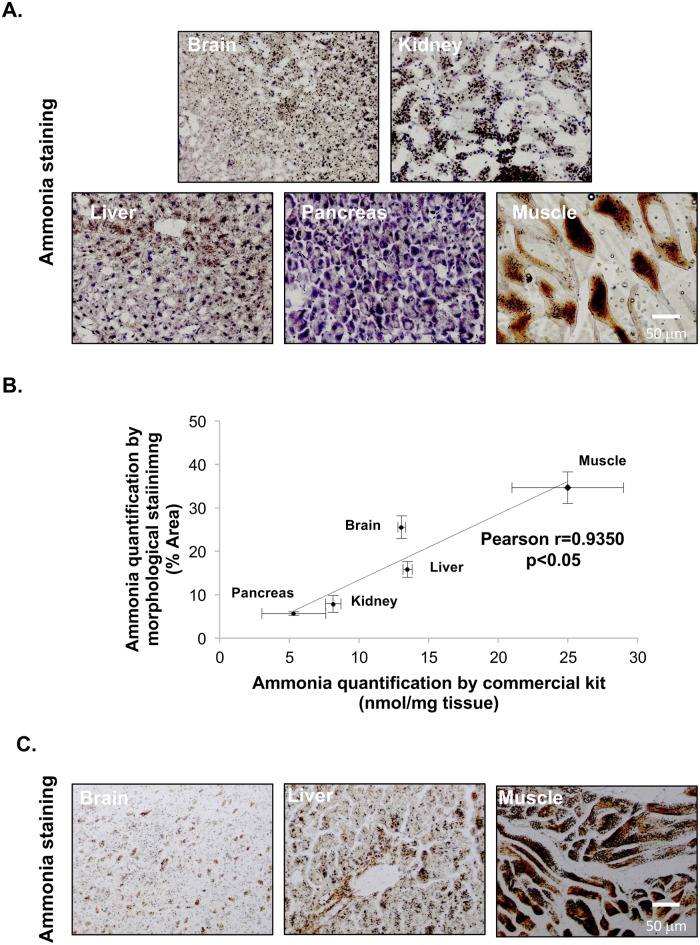
Representative micrographs of ammonia staining in healthy mouse tissue. Representative micrographs of ammonia staining in healthy mouse tissues in samples included in O.C.T **(A)** and Pearson correlation of the quantification of ammonia in biological tissues between the novel proposed method and the commercial available colorimetric kit **(B)**. Representative micrographs of ammonia staining in healthy mouse tissues in samples included in paraffin **(C)**. At least triplicates were used for each sample.

Using our morphological method, we have stained different biological tissues from healthy mice. Representative photomicrographs from O.C.T. included biological samples from different tissues are shown in Fig 1A. Coefficient of variations (C.V.) of the 5 measurements of the images of ammonia staining obtained from each sample are within 1–5% whereas the C.V. between ammonia measurements of 3 different sections obtained from the same healthy mouse liver sample is between 13–41% (n = 3), indicative of good assay precision. To further validate our method, we have quantified ammonia in fresh collected tissues from the same animals using a commercially available kit. A significant positive Pearson correlation (Pearson r = 0.9350, *p*<0.05) was found between the ammonia levels in the different organs using the two different methodologies (Fig 1B). Paraffin-embedded samples of some tissues (e.g. brain, liver and muscle) were also performed and are similar to the O.C.T. included samples, showing that both inclusion methods can be indistinctively used for ammonia determination (Fig 1C).

### Hepatic ammonia in acute and chronic liver injury

**Fig 2:**
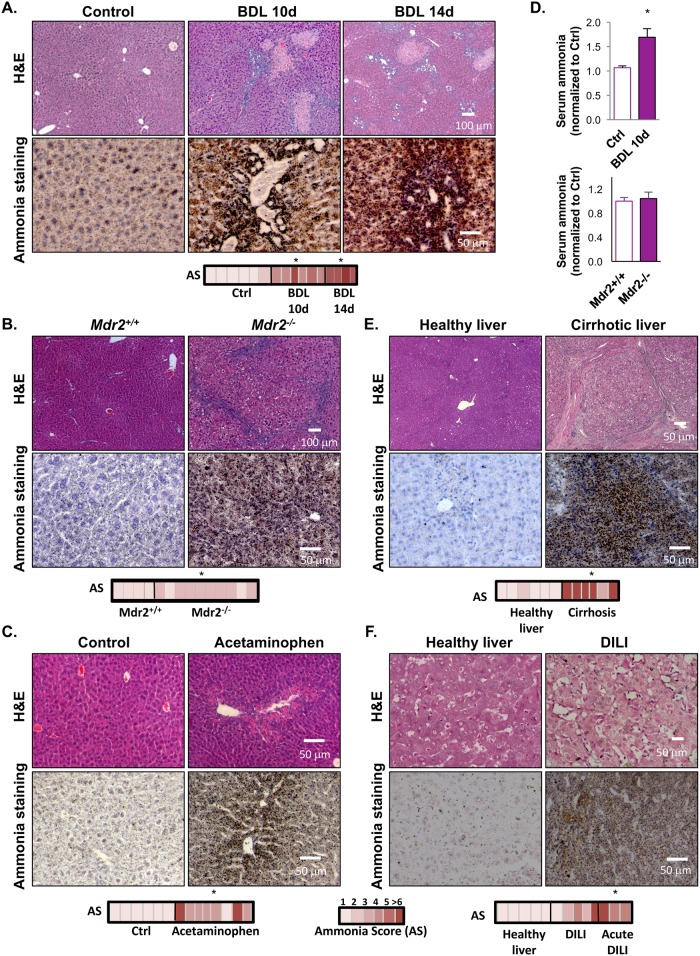
Hepatic ammonia staining in mouse models and human samples of acute and chronic liver injury. Representative micrographs of H&E and ammonia staining in liver samples in bile-duct ligation (BDL) mouse model of liver injury (n = 6 Ctrl, n = 6 BDL 10 days and, n = 4 BDL 14 days, *p<0.05 vs. Ctrl) **(A)**, in the transgenic mouse model of cholestatic disease, the Mdr2^-/-^ mouse (n = 6 Mdr2^+/+^ and n = 6 Mdr2^-/-^, *p<0.05) **(B)** and, in acetaminophen-induced liver injury in mouse (n = 6 Ctrl, n = 8 Acetaminophen, *p<0.05) **(C)**. Serum ammonia both in BDL and in the Mdr2 mouse models are shown, *p<0.05 is indicated **(D)**. Finally, representative micrographs of H&E and ammonia staining in the liver of cirrhotic patients (n = 6 healthy and n = 6 Cirrhosis, *p<0.05) **(E)** and in idiosyncratic drug-induced liver injury (DILI) patients (n = 5 healthy, n = 5 DILI and n = 3 Acute DILI, *p<0.05 Acute DILI vs. healthy) **(F)** are shown.

Ammonia accumulation is a critical step in acute and chronic liver injury [1]. To further validate our method, we have assessed ammonia accumulation in liver samples from different animal and clinical models of liver injury. In the representative micrographs from paraffin embedded liver samples shown in Fig 2A, 2B and 2C, we observe that the amount of ammonia in mouse livers, represented as AS, is progressively increased after BDL surgery, in the liver cholestasis model of Mdrd2^-/-^ mouse, as well as after 48h of acetaminophen-induced liver injury in mouse livers. Damage in liver tissues was confirmed by H&E staining. Whereas in BDL mouse model, hepatic ammonia levels are associated with increased serum ammonia, in the Mdr2^-/-^ mouse, increased liver ammonia levels are not reflected in serum (Fig 2D). This discrepancy may partly reflect the distinct role of kidney in these models, being that in the BDL mouse model, urea renal clearance is disrupted as previously described [27]. In addition, representative liver photomicrographs of H&E and ammonia stainning of either control or cirrhotic patients are shown in Fig 2E. As expected, our method clearly shows increased AS in cirrhotic samples. Likewise, samples from DILI patients have increased AS, especially in the patients whose sample biopsy was collected during the acute event (Fig 2F). These data are consistent with the finding that our method and resultant AS is sensitive to the severity of hepatic pathology.

### Hepatic ammonia in NAFLD

**Fig 3:**
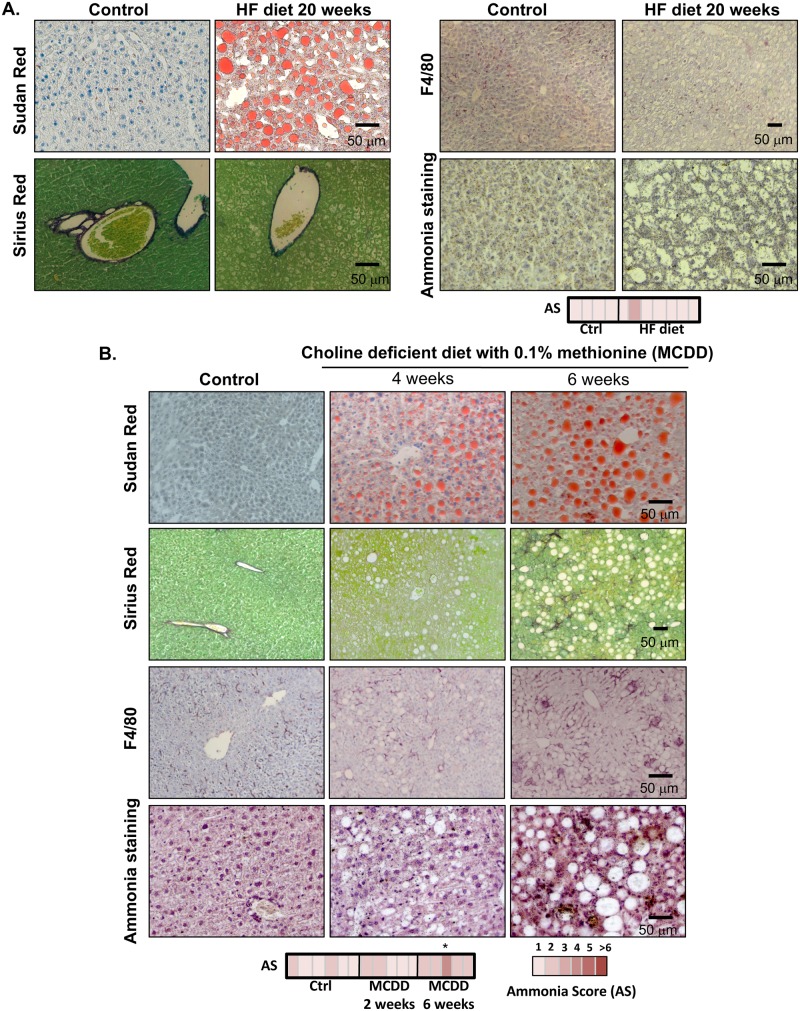
Hepatic ammonia staining in mouse models of Non-Alcoholic Fatty Liver Disease (NAFLD). Representative micrographs of Sudan Red, Sirius Red, F4/80 and ammonia staining in liver samples from high-fat (HF) diet (n = 6 Ctrl vs. n = 7 HF diet), (A) and choline deficient with 0.1% methionine diet (MCDD)-fed rodents (n = 6 Ctrl vs. n = 5 MCDD 2 weeks and n = 5 MCDD 6 weeks) (B). AS was only significantly increased in MCDD 6 weeks vs. Ctrl, p<0.05.

**Fig 4:**
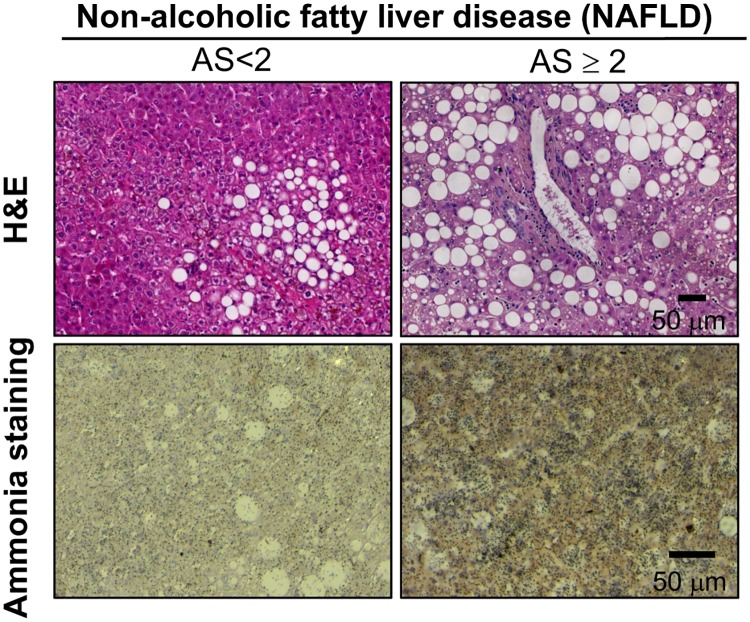
Hepatic ammonia staining in patients with Non-Alcoholic Fatty Liver Disease (NAFLD). Representative micrographs of H&E and ammonia staining in liver samples from NAFLD patients with low (<2) and high (≥2) Ammonia Score (AS).

In here, we have assessed the accumulation of ammonia in NAFLD both in animal models of NAFLD and in the clinical setting. As a result of high fat diet, animals accumulate fat in the liver in spite of the absence of overt inflammation and fibrosis. Under these circumstances, AS in these animals is not increased relative to their healthy counterparts (Fig 3A). However, when we measure ammonia in a mouse model fed a choline deficient diet supplemented with 0.1% methionine, we observed that AS is augmented after 6 weeks of dietetic regimen, a time point characterized by high degree steatosis, inflammation detected by F4/80 accumulation, a membrane macrophage marker, and mild fibrosis (Fig 3B). In addition, in a cohort of well-characterized NAFLD obese patients, multiple linear regression analysis showed that the degree of lobular inflammation (B = 1.364, p = 0.044) and plasma homocysteine (B = 0.215, p = 0.003) are independent predictors of ammonia levels adjusting for age and sex (S1 Table). Representative micrographs of NAFLD patients with low and high AS are shown in Fig 4. In summary, our data indicate that ammonia accumulation is augmented in advanced NAFLD.

## Discussion

Even though the accurate quantification of ammonia in the liver could be potentially used as a predictive marker of the outcome of patients with liver disease [17–19], the methods currently used for the quantification of hepatic ammonia are not optimized for routine clinical assay. Thereby, clinicians often defer to indirect methods of liver ammonia content such as blood or urine ammonia or a recently described approach measuring ammonia in human breath [28]. Whereas the determination of blood and urinary ammonia is very imprecise mostly due to environmental factors that affect sample quality and delays in completion of analysis [20], the determination of ammonia in human breath is a protocol still in a very early stage that remains to be validated. Furthermore, increased ammonia level in the blood may not exactly reflect hepatic ammonia content, as it mirrors whole body nitrogen metabolism, where liver, kidney and muscle also play an important role on maintaining ammonia homeostasis. As an alternative, the quantification of ammonia in liver biopsies is an option as the biopsy is often performed in patients with liver disease for accurate diagnosis. Currently, the methods used for the quantification of ammonia in biological tissues often rely on commercially available kits where ammonia extraction is coupled to colorimetric assays. These assays are rather expensive (≈15 U.S.D per sample) and sensitive to ammonia sources present in the environment being that working on a glovebox or a negative air pressure area, not always available at all the clinical facilities, is necessary. Moreover, prolonged sample storage, even at -80°C, may affect the stability of samples resulting in ammonia readings lower than expected.

Herein, we have developed a novel morphological method by using Nessler´s reagent that allows the accurate and precise quantification of ammonia in biological tissues. This method is fast (less than one hour if using O.C.T. included samples), inexpensive (about 3 U.S.D. *per* sample), requires standard laboratory equipment already present in clinical settings that routinely perform histological analysis and does not require expert pathologist analysis as it´s based on automated analysis by using Image CellProfiler open-source software. In our current work, we provide evidence that our assay can distinguish between levels of ammonia either in different tissues from a healthy rodent or to differentiate the distinguished levels of ammonia in injured liver samples. On this regard, we have covered different stages of liver injury and observed an increase in the ammonia levels in relationship with the degree of injury in the different animal models used. We have also validated our assay in a cohort of patients with cirrhosis where measurements of liver ammonia correlate well with liver disease progression. A more complete study correlating the levels of ammonia in the liver of cirrhotic patients and the patient outcome would be informative to see if the level of ammonia for example correlates with the MELD score, a score currently used to quantify end-stage liver disease for transplant planning. In addition, our method is able to provide good estimates of liver ammonia in DILI, both in animal models and clinical samples, being specially increased in acute episodes, as expected.

Moreover, by using this method we provide evidence that ammonia is increased in more severe stages of NAFLD, at least in animal models of diet-induced fatty liver. In NAFLD patients, the results are less conclusive, even though we suggest that the accumulation of ammonia is augmented in patients with increased degree of lobular inflammation and plasma homocysteine. This is interesting considering the recent report about the role of deregulated hepatic methionine metabolism driving homocysteine elevation in NAFLD [29]. Importantly, through the transsulfuration pathway, homocysteine is converted to cystathione and then cystathione γ-liase breaks down cystathione into cysteine, α-ketobutyrate, and ammonia. Furthermore, our evidence reporting ammonia accumulation in the initial stages of inflammation in NAFLD allow us to speculate about the potential role for ammonia accumulation in the progression of this disease. Taking this into account, as in here, we have mainly focused on patients with mild disease, displaying low or moderate NAS score (1<NAS score≤5), we expect that ammonia score will be significantly higher in patients showing a higher NAS score (NAS Score>6). Thus, further experiments are necessary to conclusively address the role of ammonia accumulation in fatty liver progression to NASH and cirrhosis.

In summary, we have developed and validated a novel morphological method for ammonia detection in biological tissue with a strong potential for its clinical translation.

## Supporting information

S1 Table Characterization of NAFLD patients separated according to different Ammonia Scores (AS). *p<0.01 is indicated.
(DOCX)
