# Supplementary material for: A morphological method for ammonia detection in liver
Source: PLoS One. 2017 Mar 20;12(3):e0173914. doi: 10.1371/journal.pone.0173914 (PMC5358814; doi:10.1371/journal.pone.0173914)
Supplement: S1 Table — *p<0.01 is indicated. (DOCX) [file pone.0173914.s001.docx]

|  | **AS<2** | **AS≥2** | ***p*** |
| --- | --- | --- | --- |
| **N** | 22 | 15 |  |
| **Age (Years)** | 42.7±2.3 | 45.7±7.7 | 0.468 |
| **Gender (% women)** | 59 | 41 | 0.243 |
| **BMI** | 48.6±1.0 | 49.2±2.0 | 0.746 |
| **HOMA** | 5.8±1.0 | 4.2±2.3 | 0.331 |
| **AST (U/l)** | 39.4±10.4 | 31.1±5.6 | 0.585 |
| **ALT (U/l)** | 53.8± 13.3 | 29.9±4.4 | 0.214 |
| **Plasma Homocysteine (μmol/l)** | 10.0±0.6 | 13.9±1.4 | 0.005** |
| **NAS score (0-8)** | 3.3±0.3 | 3.1±0.7 | 0.718 |

**S1 Table.** **Characterization of NAFLD patients separated according to different Ammonia Scores (AS).** *p<0.01 is indicated.
